# *BDNF* gene polymorphisms and BDNF serum concentration in schizophrenia patients: a pilot study

**DOI:** 10.3389/fpsyt.2025.1556079

**Published:** 2025-05-19

**Authors:** Anastasiia S. Boiko, Irina A. Mednova, Ekaterina V. Mikhalitskaya, Diana Z. Paderina, Dmitry A. Petkun, Elena G. Kornetova, Nikolay A. Bokhan, Svetlana A. Ivanova

**Affiliations:** ^1^ Mental Health Research Institute, Tomsk National Research Medical Centre, Russian Academy of Sciences, Tomsk, Russia; ^2^ Psychiatry, Addictology and Psychotherapy Department, Siberian State Medical University, Tomsk, Russia

**Keywords:** schizophrenia, BDNF, gene polymorphism, biomarker, neuroplasticity

## Abstract

**Objectives:**

The search for the genetic basis of the leading symptom domains of schizophrenia is of interest. BDNF is a universal neurotrophin that promotes brain development and neuroplasticity. Our aim was to study polymorphisms of the *BDNF* gene and serum levels of BDNF in schizophrenia and to analyze the concentration of this marker depending on clinical and genetic characteristics.

**Methods:**

A clinical and biological examination of 123 patients with paranoid schizophrenia (F20.0, ICD-10) was conducted. The control group consisted of 193 healthy individuals. Genotyping of polymorphisms (rs6265 and rs11030104) was performed by RT-PCR. BDNF concentration was determined using xMAP technology. Statistical data processing was performed in SPSS software.

**Results:**

A lower BDNF concentration was found in schizophrenia patients than in healthy individuals. Clinical characteristics of the disease, such as duration of the disease and leading clinical symptoms do not affect the level of BDNF. The continuous type of course is characterized by a tendency to decrease the BDNF serum concentration compared to the episodic type. The distribution of rs6265 genotypes differed significantly between the groups of schizophrenia patients and healthy individuals. The TT genotype was more common among the patients and had a predisposing effect on schizophrenia. Serum levels of BDNF did not differ between the patients with different genotypes.

**Conclusions:**

Our results support a potential value of studied BDNF protein and gene as a neurobiological marker for schizophrenia pathogenesis and clinical characteristics. Further case-control studies on the *BDNF* gene and peripheral BDNF levels with larger sample sizes and different ethnic groups are needed to better understand the pathogenesis of the schizophrenia.

## 1 Introduction

The relevance of studying schizophrenia is due to its high prevalence in the general population (among mental disorders), chronic course, and high prevalence of disability among the patients. More than 24 million people worldwide suffer from schizophrenia, according to the World Health Organization (WHO) (1). The etiology and pathogenesis of schizophrenia are not sufficiently studied, and the disease has a multifactorial nature. At present, genetic predisposition to schizophrenia and its polygenic nature are beyond doubt (2, 3); accordingly, the search for the genetic basis of this disease is of interest.

At the same time, not enough attention is paid to the mechanisms of neuroplasticity. One of members of the neurotrophin family is brain-derived neurotrophic factor (BDNF), which plays a decisive role in the growth of dendrites, branching of axons, and formation of synapses. BDNF is a universal neurotrophin that promotes brain development, neuronal survival (4), and maintenance of dendritic branching and, as a result, neuroplasticity (5). In the adult brain, BDNF shows high expression and regulates both excitatory and inhibitory synaptic transmission (6). Meanwhile, a deficiency in BDNF signaling has been found in the pathogenesis of a number of brain diseases and mental disorders, such as Huntington’s disease, Alzheimer’s disease, and depression (7, 8).

BDNF has been researched as a potential biomarker of schizophrenia. The literature data show decreased serum BDNF concentrations in comparison of healthy persons or no significant differences (9–16).

The BDNF gene rs6265 polymorphism also has been widely investigated in relation to schizophrenia, and the results remain ambiguous. Gratacòs et al. (17) in a meta-analysis of 3338 patients with schizophrenia and 4635 healthy controls reported an association of BDNF rs6265 with schizophrenia (17). By contrast, in the meta-analysis of Vajagathali and Ramakrishnan (18), no association was found between this polymorphism and the risk of schizophrenia (18).

Most studies have examined either the serum level of BDNF or the association of the disease with gene polymorphisms separately. Nonetheless, there are only a few studies (which contradict one another) that have assessed the level of peripheral BDNF and its gene polymorphism simultaneously in the same patients (19).

*The aim of the research:* to investigate polymorphisms of the BDNF gene and serum levels of BDNF in schizophrenia patients and to analyze the concentration of this marker depending on clinical and genetic characteristics.

## 2 Methods

### 2.1 Participants

This study was carried out in accordance with the Code of Ethics of the World Medical Association and complied with the Declaration of Helsinki (1975, revised in Helsinki, 2024). Each patient provided written informed consent after the study was approved by the Local Bioethics Committee at the Mental Health Research Institute of Tomsk National Research Medical Center (hereafter: Tomsk NRMC).

A clinical and biological examination was performed on 123 patients with paranoid schizophrenia (F20.0 according to ICD-10) undergoing treatment in the clinics of the Mental Health Research Institute of Tomsk NRMC and in Tomsk Regional Clinical Psychiatric Hospital.

The inclusion criteria were a verified clinical diagnosis of schizophrenia (F20) according to the World Health Organization World Mental Health Composite International Diagnostic Interview (WHO WMH-CIDI) for schizophrenia diagnostics, age 18–65 years, and the patient’s informed consent. Exclusion criteria for all patients were non-Caucasian physical appearance (e.g., Mongoloid, Buryats or Khakassians), organic mental disorders (e.g., epilepsy, Parkinson’s disease) infectious-inflammatory and autoimmune diseases or somatic disorders in the stage of decompensation and persons who used illegitimate psychoactive substances.

The severity of clinical and psychopathological symptoms was assessed using the Positive and Negative Syndrome Scale (PANSS). The PANSS scale in the adapted Russian version – SCI–PANSS was filled out by a psychiatrist upon admission of the patient to the hospital. Data were collected about baseline antipsychotic therapy and concomitant therapy at the time of examination and during the previous 6 months (medicines and doses administered, and duration of current medication use). For dose standardization, the daily dose of a chlorpromazine equivalent (CPZeq) was used.

The control group consisted of 193 mentally and somatically healthy individuals, sex-matched (with the patient group), without chronic diseases and signs of acute infectious diseases at the time of examination.

The main characteristics of the analyzed groups are presented in **Table 1**.

**Table 1:** Demographics and clinical characteristics of the study population.

Parameter	Patients with schizophrenia (*n* = 123)	Healthy persons (*n* = 193)
Age (Me [Q1; Q3]), years	38 [33; 47]	38 [30; 53]
Sex:		
Male, *n* (%)	53 (43.1%)	89 (46%)
Female, *n* (%)	70 (56.9%)	104 (54%)
Duration of schizophrenia (Me [Q1; Q3]), years	12 [5; 20]	NA
Duration of schizophrenia less than 5 years, *n* (%)	34 (27.6%)	NA
Duration of schizophrenia more than 5 years, *n* (%)	89 (72.4%)	NA
Continuous type of schizophrenia, *n* (%)	42 (40.8%)	NA
Episodic type of schizophrenia, *n* (%)	49 (47.6%)	NA
Leading symptom:		
Negative symptom, *n* (%)	66 (53.7%)	NA
Positive symptom, *n* (%)	57 (46.3%)	NA
PANSS total (Me [Q1; Q3])	99 [88; 108]	NA
Type of antipsychotics:		
Typical AP, *n* (%)	49 (39.8%)	NA
Atypical AP, *n* (%)	74 (60.2%)	NA
CPZeq (Me [Q1; Q3])	430 [267; 798]	NA

PANSS, Positive and Negative Syndrome Scale; AP, antipsychotics; CPZeq, antipsychotic dose in chlorpromazine equivalents; Me [Q1; Q3] – median and quartiles.

Blood was collected from participants in the morning on an empty stomach into tubes containing an anticoagulant (EDTA) (whole blood) and tubes with a clot activator (SiO_2_) (to obtain serum).

### 2.2 Genotyping

DNA extraction was performed by the standard phenol–chloroform method from whole blood leukocytes. The resulting samples were frozen at – 20°C. A working DNA solution was prepared from the frozen samples. Genotyping of *BDNF* gene polymorphisms (rs6265 and rs11030104) was performed using real-time PCR on a StepOnePlus Real-Time PCR System and QuantStudio 5 Real-Time PCR System (Applied Biosystems, USA) (core facility Medical Genomics, Tomsk NRMC).

### 2.3 Measurement of BDNF concentration

Blood serum was obtained by centrifugation to separate erythrocytes under standard conditions (30 min at 1500g and 4°C), after which the samples were frozen and stored at – 80°C.

The concentration of BDNF was determined in blood serum of 123 patients and 74 healthy individuals via xMAP technology on analyzers Magpix and Luminex 200 (Luminex, USA) (core facility Medical Genomics, Tomsk NRMC). For this purpose, a kit manufactured by MILLIPLEX MAP (Merck, Germany) was used. The serum BDNF concentration was measured in pg/ml, the final concentration results are presented in ng/ml. The detection results were processed in the xPONENT software (Luminex, USA) with subsequent data transfer to MILLIPLEX Analyst 5.1 software (Merck, Germany).

### 2.4 Statistical analyses

A power analysis was conducted in this study. The power of the sample was 0.95 (with a significance level of 0.05 and Cohen’s w = 0.37 (medium effect size).

Statistical data processing was performed using SPSS software, v.23 for Windows (SPSS Inc.). The data were tested for distribution type using the Kolmogorov-Smirnov test (with Lilliefors correction) and the Shapiro-Wilk test. Results are presented as Me [Q1; Q3] (median and quartiles) for data that did not follow a normal distribution. The Mann-Whitney or Wilcoxon test was used to analyze the data of the two groups. The Kruskal-Wallis test was used for multiple comparisons of samples. The modified chi-square (χ^2^) test was employed to evaluate the compliance of the genotype and allele frequency distribution of the studied genes with the Hardy–Weinberg distribution. Allele and genotype frequencies were compared between the two groups by the Pearson χ^2^ test. To assess the association of different genotypes, odds ratios (OR) with 95% confidence intervals (95% CI) were calculated. The significance level was set to 0.05.

## 3 Results

**Figure 1:**
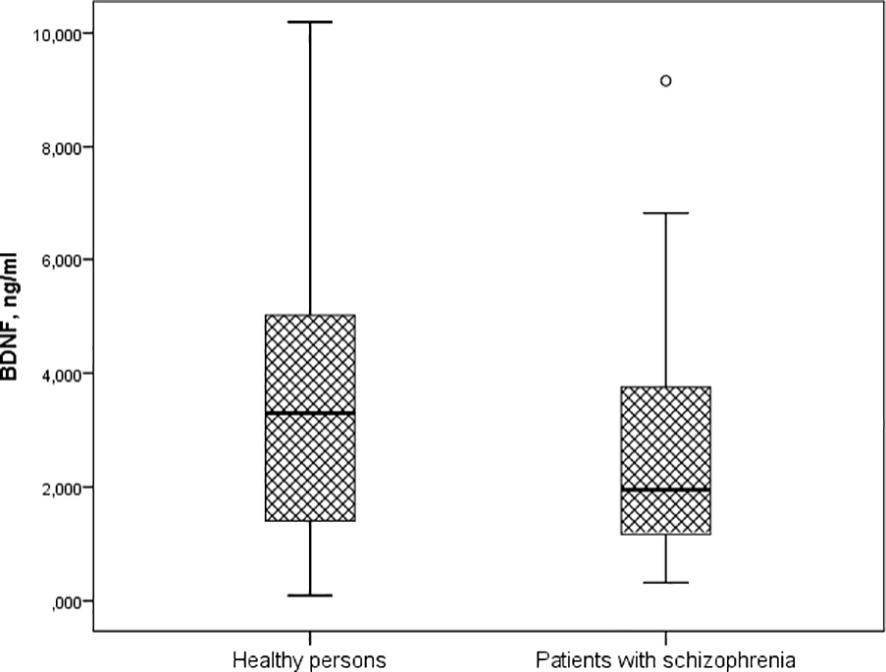
Serum BDNF levels in patients with schizophrenia and healthy persons (Me [Q1; Q3], p=0.015). Notes: BDNF – brain-derived neurotrophic factor; Me [Q1; Q3] – median and quartiles; p-value – significant differences by U-test Manna-Whitney.

A significantly lower BDNF concentration was found in patients with schizophrenia (1.5 [1.167; 3.836] ng/ml) compared to healthy individuals (3.298 [1.365; 5.044] ng/ml) (p = 0.015) (**Figure 1**).

Clinical characteristics of the disease, such as duration of the disease and leading clinical symptoms do not affect the level of BDNF. The continuous type of course is characterized by a tendency to decrease the BDNF serum concentration compared to the episodic type: 1.872 [1.078; 3.108] ng/ml and 2.723 [1.434; 4.721] ng/ml, accordingly(p=0.062).

**Table 2** presents genotype and allele prevalence rates of *BDNF* gene polymorphisms in schizophrenia patients and in the control group.

**Table 2:** The distribution of BDNF genotypes (n, %) among the patients and healthy persons.

Polymorphic variant	Genotypes/Alleles	Patients with schizophrenia (*n* = 115)	Healthy persons (*n* = 193)	χ^2^	p-value
rs6265	CC	90 (78.3%)	142 (73.6%)	7.77	0.021*
	CT	20 (17.4%)	50 (25.9%)		
	TT	5 (4.3%)	1 (0.5%)		
	C	200 (87%)	334 (86.5%)	0.023	0.88
	T	30 (13%)	52 (13.5%)		
rs11030104	AA	81 (70.4%)	133 (68.8%)	2.509	0.285
	AG	29 (25.2%)	57 (29.6%)		
	GG	5 (4.3%)	3 (1.6%)		
	A	191 (83%)	323 (83.7%)	0.042	0.838
	G	39 (17%)	63 (16.3%)		

*Significant differences at a p-value less than 0.05.

The distribution of rs6265 genotypes differed significantly between the two groups (χ^2^ = 7.77, p = 0.021). The TT genotype proved to be more common among patients with schizophrenia and had a predisposing effect on this mental disorder.

Analysis of the BDNF concentration in blood serum in relation to the carriage of genotypes of the *BDNF* gene polymorphisms in healthy persons and patients with schizophrenia was the next stage of our work. Serum levels of BDNF did not differ significantly between patients having different genotypes of the polymorphic variants under study (**Table 3**).

**Table 3:** Serum BDNF levels in relation to genotypes in patients with schizophrenia and control group.

Polymorphic variant	Genotypes/Alleles	BDNF, ng/ml	p-value*
Schizophrenia patients			
rs6265	CC	1.996 [1.167; 3.673]	0.838
	CT	1.434 [1.129; 3.914]	
	TT	2.089 [1.114; 4.336]	
rs11030104	AA	2.049 [1.158; 3.608]	0.89
	AG	1.779 [1.161; 3.966]	
	GG	2.840 [1.052; 4.908]	
Healthy persons			
rs6265	CC	3.03 [2.57; 4.69]	0.479
	CT	4.83 [1.541; 5.99]	
	TT	3.459	
rs11030104	AA	3.136 [2.616; 4.728]	0.884
	AG	4.094 [1.389; 5.276]	
	GG	3.615 [3.459; - ]	

*Kruskal–Wallis test. Me [Q1; Q3] – median and quartiles.

## 4 Discussion

In this paper, we report results of a study on possible associations of BDNF and two variants of the *BDNF* gene with schizophrenia. We found significantly lower serum BDNF levels in patients with schizophrenia than in healthy controls. Our results are consistent with the literature data that show decreased serum BDNF concentrations in both drug-naïve (9–11) and antipsychotic-treated patients (12–14). Our study, like a large number of other literary studies, was conducted in serum and the question remains open as to how adequately the determination of serum content reflects the level of BDNF in the brain and whether it is possible to talk about its role in the pathogenesis of schizophrenia. The results of studies about correlations between central and peripheral BDNF are contradictory, however, there is some evidence that the concentration of BDNF in the brain reflects serum BDNF. BDNF’s ability to cross the blood–brain barrier suggests that the BDNF levels measured in the peripheral blood may reflect its levels in the brain (20). Pillai et al. (21) demonstrated parallel changes in the BDNF levels in the plasma and CSF of patients with schizophrenia. The analisys of correlations between serum BDNF and brain volume found significant associations between serum BDNF levels and right and left hippocampal volume with lower BDNF corresponding to lower volumes (22). Thus, with certain limitations, peripheral BDNF can be considered as a biomarker of the processes of neuroplasticity, neurogenesis and neuroprotection (23, 24). Schizophrenia is a fairly heterogeneous group with different leading clinical symptoms, different types of disease progression and different duration. All these factors can have certain effects on the content of peripheral markers. We demonstrated for the first time that clinical characteristics of the disease, such as duration of the disease and leading clinical symptoms do not affect the level of BDNF. The continuous type of course is characterized by a tendency to decrease the BDNF serum concentration compared to the episodic type (p=0.062).

BDNF is certainly involved in the pathogenesis of schizophrenia, which is confirmed by data on its decrease in patients with the first episode of schizophrenia (9, 25), however, the antipsychotic therapy used also affects its level.

The role that antipsychotics play on BDNF levels is highly controversial, and there are even two meta-analyses with contradictory results regarding whether or not antipsychotic treatment corrects BDNF levels (26, 27). The results of treatment studies in human patients and animals suggest that second-generation antipsychotics compared to first-generation antipsychotics have neuroprotective effects. The animal studies indicate that these effects are probably mediated through increased expression of brain derived neurotrophic factor (28). There are differences between typical or atypical antipsychotics or even between different atypical antipsychotics with an apparent greater capacity to correct BDNF levels for the atypical ones and within them clozapine or aripiprazole (25). Antipsychotic medications appear to have distinct effects on serum BDNF levels in short-and long-term treatment and also differ in patients in acute conditions and in remission (29).

We noticed an association of the *BDNF* gene rs6265 polymorphism with schizophrenia. This polymorphism has been widely investigated in relation to schizophrenia, and the results remain ambiguous. Gratacòs et al. (17) in a meta-analysis of 3338 patients with schizophrenia and 4635 healthy controls reported an association of *BDNF* rs6265 with schizophrenia (17). By contrast, in the meta-analysis of Vajagathali and Ramakrishnan (18), no association was found between this polymorphism and the risk of schizophrenia (18). These discrepancies in results may be due to the high clinical heterogeneity of schizophrenia and differences in the carriage of polymorphisms among various ethnic groups (these differences are not taken into account in meta-analyses). Case-control studies point to an association of rs6265 (*BDNF)* with cognitive impairment, disease severity, clinical symptoms, and a response to antipsychotic therapy (30). The rs11030104 polymorphism was found to not be associated with schizophrenia in our study. In contrast to the rs6265 single-nucleotide polymorphism, not many authors have examined the association between rs11030104 and mental disorders. An association has been detected between this polymorphism and a response to antipsychotics or adverse effects of drugs (31–33). To our knowledge, our study is the first to show the absence of an association between rs11030104 and schizophrenia in white individuals.

Single-nucleotide polymorphism rs6265 is functional and results in the substitution of valine (Val) with methionine (Met) in the BDNF protein. *In vitro* experiments have revealed that this polymorphism affects activity-dependent secretion of BDNF (34). Nonetheless, we did not find any differences in serum BDNF levels between patients with different genotypes. Our results are consistent with those reported about Indian, Polish, and Taiwanese populations (35–37). In contrast, Zakharyan and Boyajyan have demonstrated that carriers of the rs6265 minor allele have low plasma BDNF levels (38). The rs11030104 polymorphism is not functional but associated with intron variants in both the *BDNF* gene and *BDNF-AS* gene (30). This observation may explain the lack of association between serum BDNF levels and genetic variants of this polymorphism in our study.

### 4.1 Limitations

Our research has several limitations. It is a pilot study which a small-scale preliminary study conducted to check the feasibility or improve the research design and attract the attention of other researchers to this topic. Symptoms were assessed during the diagnostic interview and may therefore reflect a treatment effect, as the patients studied were receiving pharmacological treatment with antipsychotic medications; thus, we cannot distinguish the association of genotype with the underlying symptom profile from its association with treatment response and the influence of pharmacotherapies on the content of BDNF. To overcome this limitation, longitudinal studies of initially medication-naïve patients would be needed or assessment of the content of BDNF in the dynamics of monotherapy with first or second generation antipsychotics. The fact that only Caucasians were included in the study limits the generalizability of the study, although we attempted to achieve ethnic homogeneity in the sample.

## 5 Conclusions

Our results support a potential value of studied BDNF protein and gene as a neurobiological marker for schizophrenia pathogenesis and clinical characteristics. Further case-control studies on the *BDNF* gene and BDNF peripheral levels with larger sample sizes and different ethnic groups are needed.

## Data Availability

The original contributions presented in the study are included in the article/supplementary material. Further inquiries can be directed to the corresponding author.
